# Implementing an Artificial Intelligence Decision Support System in Radiology: Prospective Qualitative Evaluation Study Using the Nonadoption Abandonment Scale-Up, Spread, and Sustainability (NASSS) Framework

**DOI:** 10.2196/80342

**Published:** 2026-01-28

**Authors:** Sundresan Naicker, Paul Schmidt, Bruce Shar, Amina Tariq, Ashleigh Earnshaw, Steven McPhail

**Affiliations:** 1 Australian Centre for Health Services Innovation School of Public Health and Social Work Queensland University of Technology Kelvin Grove Australia; 2 Department of Medical Imaging Princess Alexandra Hospital Woolloongabba Australia; 3 Digital Health and Informatics Directorate Metro South Health Woolloongabba Australia

**Keywords:** artificial intelligence, radiology, implementation science, decision support, qualitative

## Abstract

**Background:**

Medical imaging remains at the forefront of advancements in adopting digital health technologies in clinical practice. Regulator-approved artificial intelligence (AI) clinical decision support systems are commercially available and being embedded into routine practices for radiologists internationally. These decision support solutions show promising clinical validity compared to standard practice conditions; however, their implementation over time and implications on radiologists’ practice are poorly understood.

**Objective:**

This paper aims to examine the real-world implementation of an AI clinical decision support tool in radiology through a qualitative evaluation across pre-, peri-, and postimplementation phases. Specifically, it seeks to identify the key contextual, organizational, and human factors shaping adoption and sustainability, to map these influences using the nonadoption, abandonment, scale-up, spread, and sustainability (NASSS) framework, and to generate insights that inform evidence-based strategies and policy for integrating AI safely and effectively into public hospital imaging services.

**Methods:**

This prospective study was conducted in a large public tertiary referral hospital in Brisbane, Queensland, Australia. One-to-one participant interviews were undertaken across the 3 implementation phases. Participants comprised radiology consultants, registrars, and radiographers involved in chest computed tomography studies during the study period. Interviews were guided by the NASSS framework to identify contextual factors influencing implementation.

**Results:**

A total of 43 semistructured interviews were conducted across baseline (n=16), peri-implementation (n=9), and postimplementation (n=18) phases, comprising 7 (16%) radiographers, 20 (47%) registrar radiologists, and 16 (37%) consultant radiologists. Across NASSS domains, 56 barriers and 18 enablers were identified at baseline, 55 and 14 during peri-implementation, and 82 and 33 postimplementation. Organizational barriers dominated early phases, while technological issues such as system accuracy, interoperability, and information overload became most prominent during and after rollout. Enablers increased over time, particularly within the technology and value proposition domains, as some clinicians adapted the AI as a secondary safety check. Trust and adoption remained constrained by performance inconsistency, weak communication, and medicolegal uncertainty.

**Conclusions:**

The implementation of AI decision support in radiology is as much an organizational and cultural process as a technological one. Clinicians remain willing to engage, but sustainable adoption depends on consolidating early positive experiences and addressing negative ones, embedding communication and training, and maintaining iterative feedback between users, vendors, and system leaders. Applying the NASSS framework revealed how domains interact dynamically across time, offering both theoretical insight into sociotechnical complexity and practical guidance for hospitals seeking to move from pilot to routine, trustworthy AI integration.

## Introduction

### Background

The demand for advanced medical imaging services continues to grow at a rapid pace, and reducing time delay from image capture to radiological reporting remains a priority for public hospital services [1,2]. Radiologists often need to work through large volumes of information to report on medical images for a wide array of patient groups. This can be challenging in clinical environments with constraints on time, resources, and related workflow pressures. Excessive delay from time of medical image capture to the provision of a definitive radiological report to the referring clinical team can undermine the quality and safety of health care delivery [3,4].

Medical imaging has been, and remains, at the forefront of advancements in digital health technologies in everyday clinical practice [2,5]. Consequently, there is an array of digital tools available to support radiology decision-making that have arisen from advances in machine learning and other related technologies. Artificial intelligence (AI) algorithms that are commercially available, and with regulatory approvals already in place, are now being embedded in digital tools and readily adopted into routine practices for radiologists in various settings internationally [6,7].

In experimental and validation studies, AI algorithms show strong technical performance: meta-analyses report pooled sensitivity and specificity values exceeding 0.80-0.85 for tumor metastasis and rib fracture detection, with the mean area under the curve near 0.90 [8,9]. These results highlight the potential for AI to enhance accuracy and throughput, particularly in resource-constrained health systems [5,8,10]. Yet this technical promise has outpaced the evidence on real-world implementation [11]. The literature remains dominated by model validation [5] and cross-sectional studies of clinician trust [12-14], with few studies examining how AI systems are adopted, adapted, or sustained within the operational realities of hospital environments. Recent reviews emphasize this gap, noting that most AI-radiology research ends at performance benchmarking and fails to explore workflow integration, organizational readiness, or long-term routinization [10,15]. Broader governance and workforce analyses likewise underline persistent uncertainty around accountability, medicolegal responsibility, and system-level preparedness for AI-supported care [16,17]. Collectively, these gaps constrain understanding of how algorithmic potential translates into clinical and organizational value.

While these quantitative evaluations and meta-analyses have established AI’s diagnostic capability, they provide little insight into how and why such technologies succeed or fail once introduced into routine clinical practice. Many rely on retrospective datasets, simulated environments, or controlled reader studies that remove the influence of real-world complexity [18-21]. Consequently, they overlook the macro-, meso-, and microlevel dynamics of workflow adaptation, human-technology interaction, and organizational and sociocultural context that determine whether AI enhances or disrupts practice. A qualitative implementation approach is therefore critical for exploring the lived experiences, informal workarounds, and contextual contingencies that shape integration in situ [21,22]. Such an approach complements quantitative evidence by revealing the social and organizational mechanisms through which AI adoption is negotiated, sustained, or resisted in everyday radiology work and practice.

Emerging qualitative and mixed methods studies have begun to address aspects of these challenges by exploring radiologists’ perceptions, sources of trust and mistrust, and organizational barriers to adoption [12,23]. However, most have relied on single-time-point interviews, limited samples (n<20), or hypothetical case vignettes that do not capture the evolving interaction between users, workflows, and technology over time [18,19,24]. Few have been conducted within active service settings or have systematically linked individual experiences to organizational processes or system-level factors [12,25]. This has resulted in a descriptive but fragmented evidence base that provides limited insight into how implementation unfolds, stabilizes, or falters once AI becomes part of routine care.

This study responds directly to that gap by presenting a qualitative, end-to-end evaluation of AI implementation within a large tertiary radiology department in Brisbane, Queensland, Australia. Here, end-to-end refers to a lifecycle approach spanning predeployment context and readiness, peri-implementation adaptation, and postimplementation integration and routinization, examining how technological, human, and organizational factors interact over time [26,27]. There is a broad range of implementation frameworks to assess the implementation of a digital health innovation across a life cycle; however, they are varied in their analytical purpose [28]. Strategy-based models such as the Expert Recommendations for Implementing Change (ERIC) framework provide detailed lists of discrete implementation actions, but they are limited in explaining the mechanisms through which adoption unfolds in complex clinical environments [29]. In contrast, our evaluation sought to understand how and why AI integration succeeds or stalls within a dynamic, real-world system. The nonadoption, abandonment, scale-up, spread, and sustainability (NASSS) framework [30] was therefore selected to guide data collection and analysis. NASSS offers a theoretically grounded structure for examining the sociotechnical complexity of digital innovation by integrating the domains of technology, adopters, organization, value proposition, and wider context [31,32]. This systems-oriented lens provides greater explanatory power than more generalized implementation approaches for capturing interdependencies, contextual contingencies, and the temporal evolution of barriers and enablers across the implementation lifecycle.

By situating implementation within real-world clinical operations rather than experimental or hypothetical conditions, this study provides a rare longitudinal perspective on how AI becomes normalized or resisted within a complex hospital environment. Its findings have direct relevance to current policy efforts to scale AI responsibly in public health systems, where efficiency, safety, and governance imperatives converge [33].

### Aims

Accordingly, this paper aims to examine the real-world implementation of an AI-based clinical decision support tool in radiology through an end-to-end qualitative evaluation across pre- (baseline), peri-, and postimplementation phases. Specifically, it seeks to identify the key contextual, organizational, and human factors shaping adoption and sustainability, to map these influences using the NASSS framework, and to generate insights that inform evidence-based strategies and policy for integrating AI safely and effectively into public hospital imaging services.

## Methods

### Study Design and Theoretical Framework

This study used a qualitative prospective design. The study was structured across 3 temporal phases to capture the evolving context of AI implementation within the radiology department.

The preimplementation or baseline phase (12 months before deployment) corresponded to the period when the AI tool had not yet been introduced. This phase reflected baseline organizational conditions, established workflows, and prevailing attitudes toward digital tools in radiology.

The peri-implementation phase (an 8-week transition period) covered the initial rollout of the AI system and its integration into existing digital and reporting infrastructure. This period was characterized by early interaction with the tool and short-term adaptation of work processes.

The postimplementation phase (12 months after deployment) represented a period of stabilization in which the AI tool had become part of routine operations. This phase captured the mature context of use, reflecting how the technology was embedded, maintained, and normalized within everyday practice.

The reporting of this study was in alignment with the COREQ (Consolidated Criteria for Reporting Qualitative Studies; Multimedia Appendix 1).

### NASSS Framework

The NASSS framework was used to inform the study design and interview questions. The NASSS framework provides a systematic foundation for examining challenges across multiple domains and their dynamic interactions, which may influence the uptake, implementation, outcomes, and sustainability of technology-supported health programs [30]. It facilitates consideration of how various factors interact and adapt over time, influencing success, and includes the following domains:

1. Condition or illness: the nature and complexity of the condition being addressed.
2. Technology: the specific technology being implemented.
3. Value attributed to the technology: the perceived benefits and utility of the technology.
4. Individual adopters: the clinicians and patients using the technology.
5. Organizational adopters: the health care organizations implementing the technology.
6. External context: the broader context, including regulatory, economic, and social factors.

### Setting

This was a single-site study conducted at a large public tertiary referral hospital in Brisbane. The hospital’s Medical Imaging Department offers a comprehensive range of diagnostic imaging services to support patient care across various medical specialties.

### AI Clinical Decision Support System

The technology adopted by the department is a third-party and commercially available multiorgan AI-based computerized clinical decision support system (CDSS) for radiologists. The CDSS uses multiple specialized convolutional neural networks across the entire machine learning cycle, including preprocessing, candidate generation, classification, and postfiltering. It has been classified and approved as a diagnostic tool under the current Australian Therapeutic Goods Administration regulatory framework. The decision support system integrates with existing medical imaging hardware and software to allow computed tomography (CT) images to be automatically transferred from the scanner, preprocessed, and prepared for interpretation by radiologists. The CDSS flags or highlights any issues within the CT image that require further differential diagnosis by the radiologists. In 2021, before full site implementation, the study site tested this tool among a small group of radiologists (n=4) and anecdotally reported positive experiences.

### Participant Recruitment and Sampling

Participants included radiology consultants, registrars, and radiographers employed within the Medical Imaging Department who were involved in chest CT reporting during the study period. Recruitment was undertaken via internal email by an embedded chief investigator. A purposive yet stratified sampling approach was used to achieve broad representation across professional roles and levels of seniority. At the time of data collection, the lead interviewer (SN) was an experienced PhD-trained male implementation science researcher with no supervisory, managerial, or clinical authority over participants. SN had established professional familiarity with the department through earlier collaborative work, but no direct reporting relationships. Participants were informed of SN’s research role, disciplinary background, and interest in understanding real-world implementation challenges before interview commencement. Stratification was guided by the departmental organizational chart to ensure inclusion of participants from different functional areas and reporting responsibilities. This approach sought to capture a range of experiences across the implementation process rather than statistical representativeness. Sampling continued iteratively across the pre- (baseline), peri-, and postimplementation phases until thematic adequacy was reached, indicated by repetition of key concepts and no emergence of new issues in subsequent interviews [34].

### Qualitative Interviews

Semistructured face-to-face interviews, approximately 40 minutes in length, were conducted by an experienced implementation science researcher (SN) according to participant preference (in person, via Microsoft Teams, or through phone; Multimedia Appendix 2). All interviews were audio-recorded and transcribed upon participant consent. Interviews were conducted flexibly, with questions adapted to participant roles and experience. Not all questions or prompts were asked in every interview, but the guide provided a consistent framework to ensure coverage of key domains. The interviewer kept reflexive notes after each interview to document emerging impressions, relational dynamics, and potential influences of their positionality on data generation.

### Study Materials

We used a reflexive framework method to guide the development of a semistructured interview template, aligning with our study aims [32,35]. This approach aimed to capture a comprehensive range of insights, perceptions, and experiences, providing a rich dataset for analysis.

### Data Analysis

Interview transcripts were analyzed using an iterative, multistage process combining thematic analysis with NASSS-informed framework mapping [36-38]. Before analysis, the research team discussed their disciplinary positions and assumptions regarding AI in radiology, documenting these reflections to support analytic reflexivity. SN led coding with AE providing independent review; neither had clinical authority over participants.

Analysis and data collection were conducted concurrently to guide purposive sampling and determine saturation. Early transcript review enabled the identification of preliminary codes and gaps, informing subsequent recruitment to ensure variation in experience and role. No participant reviewed the transcripts or findings before publication, consistent with the exploratory and ecological design of the study. However, findings were discussed with senior departmental clinicians and technical leads during routine project meetings. These discussions did not involve revising data or themes but served to ensure that the interpretations accurately reflected the broader organizational context and the realities experienced within the department. This informal sense-checking supported contextual validity while maintaining analytic independence.

Initial inductive coding was undertaken by one researcher (SN), who read each transcript line by line and assigned short descriptive phrases summarizing perceived barriers, facilitators, or neutral factors related to AI implementation. To strengthen analytic credibility, a second researcher (AE) independently reviewed a subset of transcripts and the draft codebook. Coding discrepancies and interpretive differences were discussed and resolved through consensus, providing a form of cross-checking without imposing rigid interrater reliability metrics. Iterative discussions across the research team further refined code boundaries, ensuring conceptual coherence and maintaining an audit trail of analytic decisions.

Once inductive coding was complete, higher-order categories were developed to capture recurrent concepts and relationships. These categories were then deductively mapped to the NASSS framework. This process enabled systematic classification of determinants by domain (eg, technology, organization, value proposition, adopters, wider system, and clinical context) while retaining sensitivity to context-specific nuances. The mapping process was iterative, with subthemes revisited and refined to ensure conceptual alignment between inductive insights and NASSS constructs, and to account for determinants that spanned multiple sociotechnical domains. Mapped subthemes were subsequently synthesized into a set of higher-order, cross-cutting determinants representing the dynamic interactions between technological, organizational, and adopter-related factors across implementation phases. This synthesis informed the structure of the results, where inductive findings and NASSS categories are integrated to illustrate how determinants evolved from baseline to peri-implementation and into routine use. Illustrative quotes were selected by consensus to exemplify the range of perspectives within each theme and subtheme. Quote selection focused on demonstrating variation, depth, and temporal evolution rather than providing isolated examples, ensuring that quotations functioned as analytic evidence and supported the integration of inductive insights with NASSS domains. An accompanying summary table provides an at-a-glance depiction of how inductive themes aligned with specific NASSS domains and subdomains, further enhancing analytic transparency and coherence.

To enhance analytic transparency, a content count of all coded barriers and enablers was compiled in Microsoft Excel and stratified by implementation phase (baseline, peri-implementation, and postimplementation). This numerical summary illustrated the distribution and relative prominence of determinants across NASSS domains (eg, technological, organizational, and adopter-related), providing a structured complement to the qualitative narrative. The count functioned as a descriptive aid to visualize patterns within the dataset, highlight areas of convergence and divergence across phases, and support the organization of complex, multilevel determinants [39].

### Trustworthiness

To ensure trustworthiness, the research team engaged in continuous reflexive discussions throughout data collection and analysis, critically examining how their disciplinary backgrounds and assumptions could shape interpretation. Coding decisions were documented in an evolving analytic log, forming an audit trail that supported transparency and replicability. Regular peer debriefings were held to resolve interpretive differences and refine theme definitions. Trustworthiness was further reinforced through the systematic application of the NASSS framework, which provided a theoretically grounded lens for organizing inductive findings. The inclusion of a quantitative content count of coded determinants added descriptive transparency, demonstrating how interpretations were anchored in the underlying data distribution. Together, these strategies strengthened the credibility, confirmability, and dependability of the qualitative findings [35,38].

### Ethical Considerations

The Human Research Ethics Committee granted ethical clearance for this research (HREC/2021/QMS/81483). All participants provided written and verbal informed consent before participating in the study. Participation was voluntary, and participants could withdraw at any time. Participants were assured that their responses would be confidential, would not be shared with departmental leadership in identifiable form, and would have no bearing on workplace evaluation or progression. No incentives were offered, and no previous personal relationships existed between the researcher and participants beyond professional familiarity.

## Results

### Participant Characteristics

A total of 43 one-on-one interviews were conducted across the study timeframe, as shown in Table 1. This consisted of 7 (16%) radiographers, 20 (47%) registrar radiologists, and 16 (37%) consultant radiologists. A total of 9 (21%) participants were interviewed across multiple time points, consistent with public health services experiencing regular staff rotation, shift-based work patterns, and competing clinical pressures. While this posed practical challenges to longitudinal participation, it was also indicative of practical challenges with AI implementation in hospital medical imaging departments. To accommodate this and maintain the integrity of the analysis, each interview was treated as a discrete data point. This allowed us to capture a wider range of perspectives from across the workforce and reflect the dynamic, high-turnover environment typical of public hospital settings.

**Table 1:** Participant characteristics across the 3 implementation phases.

Participants		Baseline	Peri-implementation	Postimplementation
Total number of participants (N=43), n (%)		16 (37)	9 (21)	18 (42)
**Profession and seniority, n (%)**				
	Radiographer (N=7)	4 (57)	1 (14)	2 (28)
	Radiology registrar (N=20)	7 (35)	4 (20)	9 (45)
	Consultant radiologist (N=16)	5 (31)	4 (25)	7 (44)
**Sex, n (%)**				
	Male (N=26)	9 (35)	6 (23)	11 (42)
	Female (N=17)	7 (41)	3 (18)	7 (41)

### NASSS Informed Barriers and Enablers of AI Implementation in Radiology

**Figure 1:**
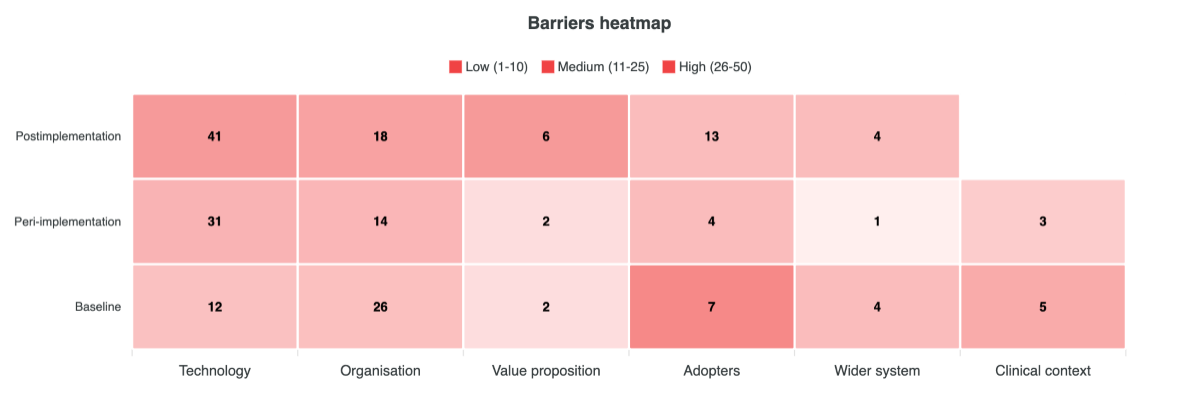
Barriers across nonadoption, abandonment, scale-up, spread, and sustainability (NASSS) domains and study phases.

**Figure 2:**
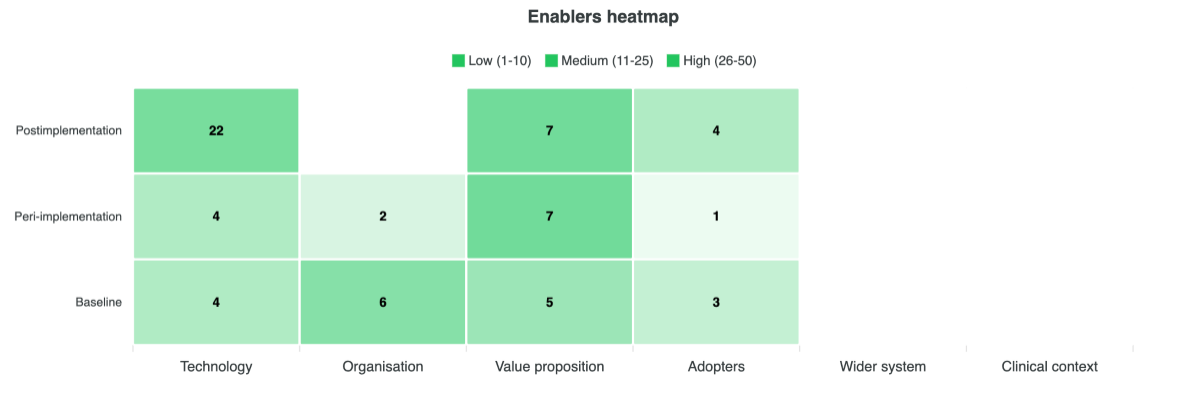
Enablers across nonadoption, abandonment, scale-up, spread, and sustainability (NASSS) domains and study phases.

A total of 56 barriers and 18 enablers were identified at baseline, 55 barriers and 14 enablers during peri-implementation, and 82 barriers and 33 enablers at postimplementation. Figure 1 presents barriers across NASSS domains and study phases, while Figure 2 shows enablers across the same phases.

At baseline, organizational barriers were the most prominent, representing nearly half of all identified barriers (26/56, 46%). These are primarily related to limited technological readiness, insufficient training, and inadequate workflow planning for implementation. Technological barriers followed (12/56, 21%), reflecting early concerns about AI performance and output accuracy, while adopter-related barriers (7/56, 12%) centered on uncertainty regarding medicolegal accountability when using AI in reporting. The main enablers at this stage were found within the organizational (6/18, 33%) and value proposition (5/18, 28%) domains, reflecting a collegial, innovation-friendly culture and a belief in the technology’s potential for efficiency and time savings.

During the peri-implementation phase, technological barriers dominated (31/55, 56%), particularly those concerning interoperability and system performance. These were followed by organizational barriers (14/55, 25%) related to weak implementation planning and inadequate workflow support, and a smaller set of adopter barriers (4/55, 7%) linked to limited trust in the AI system. Despite these issues, several enablers emerged, most notably within the value proposition domain (7/14, 50%), where participants anticipated potential efficiency gains if technical and integration challenges could be addressed. A smaller number of enablers (4/14, 28%) related to technology, as some users began using the AI system to cross-check their own interpretations.

By postimplementation, technological barriers persisted (41/82, 50%) as problems with accuracy, reliability, and speed remained unresolved. Organizational barriers (18/82, 22%) continued to reflect deficiencies in communication, training, and workflow integration, while adopter barriers (13/82, 16%) indicated ongoing distrust in the AI and reluctance to incorporate it fully into routine practice. However, this phase also saw the most substantial growth in enablers (a total of 33), particularly within technology (22/33, 67%), as users adapted the system for use as a secondary check or safety mechanism. Additional enablers were identified within the value proposition (7/33, 21%), where participants recognized relative efficiency benefits, and among adopters (4/33, 12%) who expressed emerging, albeit cautious, trust in the AI’s evolving role.

Across all NASSS domains, implementation was characterized by an interplay between anticipated risks, such as workflow integration and information overload, and realized challenges during peri-implementation, many of which persisted into routine use (Figure 1). While optimism and perceived value remained for some, trust and adoption were undermined by ongoing performance and communication barriers. Together, these patterns illustrate how implementation unfolded within a large, dynamic clinical service, with determinants shifting as the AI system moved from anticipation to early use and then attempts to move into routine practice. The relative prominence of technological, organizational, and adopter-related factors at each phase provides a contextual frame for understanding the subsequent themes. These distributions therefore situate the qualitative findings within the broader organizational and technological environment in which the AI was being implemented.

### Framework Analysis and Narrative Synthesis of NASSS Domains and Subdomains

Table 2 presents a synthesis of inductive themes mapped to the NASSS framework, illustrating how key implementation dynamics evolved across baseline, peri-implementation, and postimplementation phases. The table highlights temporal shifts in organizational readiness, technological integration, value perception, adopter engagement, and wider system influences. These findings are further expanded in the results narrative synthesis, offering deeper insight into the contextual and temporal nuances of implementation.

**Table 2:** Mapping of inductive themes to nonadoption, abandonment, scale-up, spread, and sustainability (NASSS) domains and subdomains with indicative change over time.

NASSS domain	Subdomain	Inductive theme	Change across phases
Organization	Work needed to plan, implement, and monitor change	Sustained implementation planning	Limited formal planning at baseline; reactive coordination during rollout; structured monitoring and formalized training emerged post implementation.
Organization	Organizational readiness and capacity to innovate	Relational engagement and communication	Collegial but fragmented culture at baseline; weak interteam communication during rollout; some shared ownership and cross-functional coordination developed post implementation.
Organization	Extent of change to organizational routines	Workflow optimization	Anticipated efficiency at baseline; workflow disruption and duplication during rollout; individual adaptations post implementation.
Technology	Knowledge generated	Extraneous data and information overload	Anticipated clutter at baseline became a central frustration during rollout; selective filtering and cognitive habituation emerged post implementation.
Technology	Material properties	System performance and material integration	Early confidence gave way to concerns about specificity, lag, and reliability during rollout; partial refinement occurred postimplementation, though misalignment persisted.
Value proposition and clinical context	Demand-side value	Perceived benefits for workload and safety	Strong optimism at baseline; mixed experiences during rollout; perceived value became pragmatic and context-dependent post implementation.
Value proposition and clinical context	Supply-side value	Credibility and clarity of purpose	Limited understanding of purpose and benefit early on; evolved into clearer but modest recognition of niche utility post implementation.
Adopters	Role and identity	Professional positioning and negotiated use	Curiosity and willingness at baseline; trust declined during rollout due to inconsistency and false positives; cautious, selective engagement stabilized post implementation.
Adopters	Role and identity	Learning and preparedness	Informal self-learning predominated during rollout; structured and ongoing AI^a^ literacy training emphasized post implementation.
Wider system	External context	Medicolegal uncertainty and system-level guidance	Policy and liability ambiguity persisted across phases; postimplementation reflections expanded to ethical and regulatory considerations.

^a^AI: artificial intelligence.

### Organization

There were 3 inductive subthemes that mapped under the organization domain. There were challenges with sustained implementation planning, which mapped under the NASSS subdomain of “work needed to plan, implement, and monitor change;” relational engagement and communication, which mapped to “organizational readiness and capacity to innovate;” and workflow optimization, which mapped to “extent of change needed to organizational routines.”

### Work Needed to Plan, Implement, and Monitor Change (Sustained Implementation Planning)

At baseline, participants expected limited planning and support for rollout, reflecting past experiences with digital systems. As one consultant noted:

You don't actually discover issues or problems with that new process or software or whatever until you're using it, and then often there's a lack of support on a day-to-day kind of basis.

P4, Consultant

Feedback mechanisms were also described as weak, with another adding:

There’s no way for us to feed…I don’t know of a way for me to feed that back.

P1, Consultant

These comments illustrated low confidence in the organization’s ability to anticipate or respond to implementation challenges.

During peri-implementation, radiologists described minimal systematic planning or training.

Not training per se, I think there was one meeting where they said that it was being implemented.

P20, Registrar

Another reflected:

Not very well (when asked about implementation planning)…they haven't really. Besides telling us that we're going to put it into practice, yeah, there's just not much that they're saying about it.

P22, Registrar

Such experiences made participants cautious about the department’s readiness to adopt AI, with one consultant admitting:

I think in retrospect, we could have done more in terms of educating people.

P21, Consultant

Postimplementation reflections reinforced these concerns, highlighting the ongoing absence of structured improvement strategies to support uptake and sustainment. Participants were critical of the ad-hoc implementation process, arguing that:

If there are programs that are clinically usable and are planned to be rolled out within the department, then I think it makes sense for everyone to have formal training.

P33, Registrar

Even so, there was a strong appetite for more structured professional development in AI tools, with one consultant remarking:

I would like to be taught. I am a better learner if I’m taught.

P32, Consultant

Across phases, participants viewed planning and monitoring as reactive rather than anticipatory. Despite enthusiasm for AI, the lack of systematic preparation and ongoing learning opportunities constrained the department’s ability to embed change effectively. Organizational challenges were compounded by high staff turnover and rotating clinical rosters, which limited continuity of learning and reduced opportunities for cumulative familiarity with the system. These shifting workforce conditions shaped how planning gaps were experienced and helped explain some variation in engagement and confidence across the implementation period.

### Organizational Readiness and Capacity to Innovate (Relational Engagement and Communication)

At baseline, participants described a workplace that was broadly supportive of new ideas but slow to coordinate change due to limited capacity. As one consultant noted, “in public systems, people just tend to put up with inefficiencies” (P1, Consultant). Such reflections suggested that innovation was encouraged in principle but rarely matched by structured communication or system support.

During rollout, participants described poor communication and limited coordination between teams.

Having [vendor redacted] in one corner, radiologists in another, and us talking together… takes resources to get everyone together.

P23, Radiographer

Another reflected postimplementation, “We didn’t actually tell most radiographers this was happening” (P41, Radiographer), highlighting the persistence of siloed and reactive coordination. Participants attributed emerging resistance partly to this fragmentation, explaining that “a lot of stuff was happening in the background with the PAX guys and the software people” (P43, Consultant).

Lack of early adopter involvement and unclear lines of responsibility were seen as weakening organizational readiness, even where enthusiasm for innovation remained high. This persisted into postimplementation, undermining the department’s sociotechnical capacity to support AI integration. As one consultant explained:

One of the issues is that people who understand computers are not the people who understand medicine, and vice versa. So, there’s probably a communications issue.

P29, Consultant

Participants linked these challenges to the organization’s limited capacity to learn from implementation, with one concluding:

We could have done more work with the implementation initially; there could have been more clinician involvement.

P43, Consultant

Across phases, the organization was perceived as open to innovation but constrained by weak communication channels and reactive coordination. Participants emphasized that the capacity to innovate depended less on enthusiasm than on the presence of structured dialogue, feedback loops, and shared ownership across clinical and technical teams. These relational and coordination challenges intersected with changing workload pressures and fluctuating departmental priorities, reinforcing that uptake was influenced not only by communication structures but by the broader organizational environment in which teams were continuously reconfigured.

### Extent of Change Needed to Organizational Routines (Workflow Optimization)

Across phases, participants described the introduction of the AI tool as requiring substantial adjustments to established reporting routines. At baseline, senior radiologists viewed it as a potential aid to workflow optimization, especially in easing registrar workloads. As one consultant put it:

If we were able to create a system or facilitate more report completion… particularly for the registrars, that would increase satisfaction.

P1, Consultant

This optimism reflected expectations that automation would streamline repetitive elements of reporting rather than disrupt them.

During peri-implementation, participants described the tool as introducing extra steps and interruptions to normal work patterns. One consultant explained:

It does add to the amount of things you look at… you’ve got to report your CT as normal, and then you’ve got a bunch of other sequences to scroll through at the end. Not that it adds a lot, but yeah, it does… there’s more… people have been like, what’s all these extra images? They don’t really know what to do with it too much yet either.

P18, Consultant

This illustrates a lack of clear guidance or established routines for how to use or interpret the additional studies. This persisted at postimplementation:

I was sort of holding on to reports for a few hours before signing off because I didn’t want additional data to come through that I hadn’t looked up before signing off.

P30, Consultant

Instead of reducing workload, the new process created pauses, re-checks, and deferred sign-offs. Registrars similarly described difficulty maintaining rhythm and concentration, explaining that:

It’s hard to get a routine…you have to have a different routine in your workflow for a particular assessment.

P26, Registrar

These comments captured how the tool altered the flow of image review and report finalization, requiring constant recalibration of familiar sequences. Some radiologists had developed compensatory strategies to manage these disruptions; reordering tasks, batching reports, or consciously ignoring low-yield prompts. As one consultant reflected:

So for differentials, I go back to the normal scan because the tool obscures some details. So, it’s more I use it for identifications…. So, for me, it's more identifying. Ok, there's something there. I need to go back and check (normal scans).

P31, Consultant

Overall, participants described workflow change as cumulative and largely unplanned. The tool demanded continuous microadjustments rather than a one-time shift in practice. What emerged was a pattern of individual adaptation rather than coordinated redesign; clinicians modified existing routines to fit the tool rather than the tool being aligned with the established clinical workflow. The extent of workflow disruption experienced by clinicians also reflected the realities of a service marked by rotating staff, shifting caseloads, and variable daily pressures.

### Technology

The NASSS technology domain is mapped with 2 inductive subthemes: extraneous data and information overload, aligning with the subdomain knowledge generated, and system performance and material integration, aligning with the subdomain material properties. Together, these themes captured how the technical characteristics of the AI tool shaped user experience, trust, and perceived value across implementation phases.

### Knowledge Generated (Extraneous Data and Information Overload)

At baseline, a consultant anticipated risks of information overload based on previous exposure to commercial AI tools. One explained, “You could spend all day circling these things” (P5, Consultant), capturing early concerns that automated outputs might flood readers with marginal or irrelevant findings.

During peri-implementation, these concerns materialized as the system generated excessive, low-value information:

Too much data… You really want a traffic-light system.

P25, Consultant

Another registrar observed:

I'm not sure how many people look at it. It spits out so many images and random tables

P20, Registrar

These reactions pointed to an emerging pattern of signal-to-noise imbalance, where radiologists spent more time filtering artefacts than interpreting meaningful results. By postimplementation, some users described partial adaptation, learning to disregard redundant data or mentally triage the AI’s output.

It gets a little complicated when it picks up things that are artifacts. But yeah, I can work around it.

P43, Consultant

However, frustration persisted among others who saw the clutter as undermining efficiency rather than enhancing it:

It’s a waste of time. It’s just clutter, you know? … I usually ignore it.

P37, Consultant

Across phases, information overload remained one of the most salient barriers to adoption. While individual users developed coping strategies, these adaptations reflected workaround behavior rather than genuine integration, reinforcing perceptions that the AI’s knowledge output was not yet aligned with clinical reasoning or workflow needs.

### Material Properties (System Performance and Material Integration)

Performance concerns were a defining feature of the AI’s reception, particularly during peri-implementation. A registrar characterized it bluntly as “Not very accurate. Just a splatter approach” (P17, Registrar), reflecting the perception that the system detected excessive findings without adequate specificity. Such errors eroded trust and reduced the incentive to incorporate its output into reporting routines.

By postimplementation, participants expressed more nuanced but still divided views. Some regarded the system as useful for reassurance or cross-checking:

Used it more like a check-off — especially when you have things that are complex, and there are a lot of findings.

P30, Consultant

Others found the persistent false positives distracting and demoralizing. As one put it:

For me to waste time looking at it…it’s circled this fecal matter in the splenic flexure.

P28, Consultant

Several participants emphasized that perceived technical performance shaped how often they engaged with the system at all. When lag, sensitivity issues, or interface friction increased, clinicians tended to bypass or ignore the tool. Over time, its role shifted from active decision aid to optional background reference, indicating a decline in both trust and functional value.

Interoperability problems surfaced most clearly during peri-implementation, where users described limited integration between the AI software, picture archiving and communication system (PACS), and reporting systems. One consultant explained:

That’s high-level stuff, right? That’s integrating the processing, postprocessing software with the reporting software. But we don’t have that capacity.

P21, Consultant

They further highlighted redundancy and excess image sets, noting “way too, way too many sequences…we need to distil that down.”

By postimplementation, interoperability was less salient; some technical issues with integrating the AI into the system appeared resolved, but residual inconsistencies persisted. As one registrar noted:

There’s not… uniformity to the sequences that are made. The order that they come out, …that’s different from scanner to scanner.

P26, Registrar

Availability also varied:

It’s not always there. So, you’ve got to sort of remember…to look for.

P26, Registrar

Display and PACS constraints continued to affect use:

I don’t like the way that gets displayed…that’s a PACS system…how the series [are] actually displayed.

P26, Registrar

Across phases, the material outputs of the AI, its sensitivity, specificity, and responsiveness, directly influenced its perceived usefulness. Participants consistently linked suboptimal performance to disengagement, showing that successful technological integration required not just accuracy, but reliability, responsiveness, and design alignment with radiologists’ expectations of diagnostic precision. Furthermore, while major interoperability barriers had eased, the system never fully aligned with the routine reporting infrastructure, leaving incompatibilities.

Across all technology-related subdomains, participants described a gap between what the AI produced and what clinicians could *use*. Information overload and variable system accuracy combined to erode trust and limit engagement. While technical adaptation occurred at the individual level, collective integration into practice remained constrained, signaling that technological refinement and interpretability are prerequisites for sustained adoption.

### Value Proposition and Clinical Context

Across all phases, discussions of value proposition were less prominent than those of technology or organization, but two inductive subthemes mapped clearly to the NASSS value proposition domain: perceived benefits for clinical workload and safety, which mapped to demand-side value, and credibility and clarity of purpose, which mapped to supply-side value. These intersected closely with the evolving clinical context, in which fluctuating workload pressures and infrastructure challenges shaped how the AI’s value was interpreted.

### Demand-Side Value (Perceived Benefits for Clinical Workload and Safety)

At baseline, participants viewed the AI as a potential solution to workload strain and reporting delays. Anticipated benefits were framed around efficiency, redistribution of tasks, and registrar support, signaling early optimism that automation would enhance throughput and safety. The department’s intense workload and frequent interruptions reinforced this demand-side appeal: as a consultant noted, AI might “make our job easier” (P1, Consultant).

During peri-implementation, optimism gave way to more conditional appraisals. While some identified benefits for prioritization, “It highlights a few cases that you can look at first. That’s useful when there’s a backlog” (P19, Registrar), others described it as “unreliable at the moment” (P17, Registrar). Shifts in the clinical environment also tempered expectations, staffing improved, and backlogs eased. By the postimplementation phase, perceptions of value became pragmatic and evidence-driven. Clinicians viewed the AI as a limited but occasionally useful decision support tool:

I’ve usually written my report before I look at this, and I don’t tend to change the report… it’s another look, I wouldn’t think of it as more than that

P29, Consultant

Concerns over cost-efficiency persisted:

If it was free, ambivalent… If it’s significant amounts of money… I don’t see the value because it’s more work than less.

P29, Consultant

Across phases, expectations regarding the AI shifted from broad hopes of efficiency to a more divided assessment. Some saw modest contributions to safety and prioritization, while others viewed the system as duplicating effort rather than providing genuine workload relief.

### Supply-Side Value (Credibility and Clarity of Purpose)

At the same time, participants reflected on supply-side value, questioning how clearly the system’s purpose and evidence base had been articulated.

It just gives you pictures with circles. I’m not sure what the end use is meant to be.

P20, Registrar

By postimplementation, participants had a clearer understanding of what the AI could do but remained unconvinced of its overall value. However, there was also recognition that the AI was credible in concept but still immature in delivery.

It’s getting clearer now what it could be for, but it needs to evolve. Right now, it’s still just identifying, not interpreting over time.

P43, Consultant

Some viewed potential uses to optimize efficiency and workflow, with modifications:

It could identify which studies need to be reported first…or give us measurement readings.

P46, Consultant

Maybe some of those sorts of irritations around AI could be changed, you know, or fine-tuned.

P25, Consultant

These reflections indicated that perceptions of supply-side value were prospective, anchored in what the technology could deliver if optimized, rather than what it had yet achieved.

Across phases, the vendor narrative of innovation and efficiency had not yet translated into tangible or demonstrable benefit for clinicians or the wider health system. Participants viewed the AI as promising but still lacking the evidence and clarity needed to support confident investment or large-scale deployment.

### Adopters

The NASSS adopter subdomain of role and identity is mapped with 2 inductive subthemes: professional positioning and negotiated use, and learning and preparedness. Together, these described how clinicians positioned AI within their expertise and accountability, and how limited exposure and training shaped trust, confidence, and uptake. Across phases, adoption reflected an oscillation between curiosity and skepticism, with trust becoming the key mediating factor.

### Role and Identity (Professional Positioning and Negotiated Use)

At baseline, radiologists expressed a guarded willingness to engage with AI framed less as enthusiasm and more as a professional obligation.

I think I would use it…it would be almost negligent not to look at it.

P6, Registrar

Consultants saw potential for practical support:

It could certainly help you prioritize what you are watching and what order you report things.

P8, Consultant

During peri-implementation, practical experience unsettled this cautious trust. Registrars described false positives, excessive outputs, and low sensitivity:

It’s too junior at the moment.

P17, Registrar

Trust eroded not from resistance to innovation, but from inconsistency between the AI’s promise and its performance. Clinicians voiced a recurring sentiment that while AI might one day assist safety, it currently distracts from clinical focus:

If the volume of data presented is overwhelming, then that’s negative…the strength would be as a safety net for subtle findings, not changing an overall clinical picture.

P19, Registrar

Reflecting on their initial use of the tool during peri-implementation, a registrar noted:

“It was picking up stuff that wasn’t nodules…I still had to go back and look at the images again.

P34, Registrar

By postimplementation, there was selective use and partial trust.

I look through the nodules myself first and then correlate with the software to see whether it is congruent with what I’ve come up with.

P42, Consultant

Others disengaged entirely:

It slows you down because you have to verify each little dot.

P37, Consultant

A senior consultant likened the AI to “the registrar with clever ideas, but they’re all wrong” (P40, Consultant), useful for prompting review, yet unreliable without human correction.

Trust also intersected with medicolegal anxiety. Several raised uncertainties about accountability and liability:

If the software makes a mistake, who is liable—the vendor or the radiologist? We still haven’t ironed it out.

P34, Registrar

This uncertainty reinforced their instinct to retain manual control. As a consultant observed:

If I reported every possible little dot in the chest, I’d end up with a report ten pages long, which nobody would ever read.

P37, Consultant

The line between cautious trust and defensive practice remained thin.

### Role and Identity (Learning and Preparedness)

Training and readiness remained persistently underdeveloped. During peri-implementation, there was no structured orientation or clear introduction to the system. Learning was largely self-directed and reliant on peer exchange.

Personally, I don’t think I’ve had any formal sit-down with it… I’ve just figured it out.

P19, Registrar

A vendor demonstration was held midphase, but not all clinicians attended, and some felt it was disconnected from practical workflow.

I just met the software without gathering any prior information about what this new software is.

P34, Registrar

Without clear instruction or transparency about performance parameters, early experiences became a process of trial and error rather than guided adoption, reinforcing skepticism instead of trust. By postimplementation, clinicians explicitly called for structured and continuous AI education embedded within clinical and professional frameworks.

If that is incorporated into our routine…every month we have our session doing AI cases.

P32, Consultant

Others stressed the need for broader institutional responsibility:

We are severely lacking in training with AI…it should be an integral, assessed part of our training program.

P34, Registrar

These calls reflected not only a desire for technical competence but also a wish to rebuild confidence and ensure medicolegal clarity, positioning AI as a tool that must be professionally standardized, not individually improvised.

Ultimately, clinicians saw AI competence as a new layer of professional literacy, necessary to protect judgment, maintain accountability, and engage critically with emerging tools. Their learning needs were not purely technical but ethical and epistemic: how to weigh evidence, interpret probability, and remain vigilant in an era of shared decision automation.

### Wider System

Participants described the wider system as a persistent barrier across phases. This domain reflects the external political, policy, and institutional forces, such as regulation, professional guidance, legislative, and funding models that define the environment in which implementation occurs, but which local teams cannot directly control. While wider system themes were present across phases, they did not dominate every interview, and some subissues (for example, explicit references to legislation) appeared only sporadically.

At baseline, consultants depicted a public system tolerant of inefficiency and difficult to influence. Funding constraints were raised in the context of competing pressures.

Q-Health…there’s not much money around for these sorts of things.

P1, Consultant

During peri-implementation, registrars and consultants highlighted gaps in professional guidance and medicolegal expectations.

Training/communication…college says we need to learn AI, but little practical guidance…site-to-site differences.

P24, Registrar

Others wanted clearer, proactive communication from professional bodies.

College exposure is low.

P27, Registrar

Medicolegal norms were seen to expand review obligations when AI added extra views.

Mentality in radiology, if it’s on a screen, you have to comment on it, and medico-legal, if presented, we must review everything.

P21, Consultant

By postimplementation, some brought up broader ethical and policy concerns about data provenance and the social license concerns about using it:

There is concern about the way the data is being used…if all these algorithms are being trained on everyone's data, it should be open source…it’s everyone’s.

P42, Registrar

Participants contrasted public and private system incentives and capacity, linking retention and deployment choices to wider economics and case-mix.

Public keeps me for complex cases, teaching, and feedback; private pays double.

P28, Consultant

Public vs private…pay and tech are better in private; public has collegiality and case mix.

P42, Registrar

Across phases, the wider system was characterized by limited policy levers and still developing structural and legislative readiness to support the rapid integration of AI into acute health care. Taken together, these wider-system influences interacted with internal organizational dynamics, staffing fluctuations, workload variability, and shifting operational priorities to shape the evolving trajectory of implementation across phases.

## Discussion

### Principal Findings

This study reports a prospective, qualitative, end-to-end evaluation of implementing an AI-driven clinical decision support system in a public radiology department, structured through the NASSS framework [30]. By mapping barriers and enablers across domains and phases, the study captures how early expectations shaped adoption, how sociotechnical challenges emerged during the rollout, and how these dynamics influenced long-term integration and adoption. Findings highlight that successful AI adoption depends not only on technical capability but on the alignment of organizational readiness, workflow design, and professional trust. Implementation success was governed by the interaction of multiple NASSS domains, which included interdependencies among technology, organization, adopters, and value rather than any single factor.

Weak planning and limited feedback structures (organizational barriers) amplified adopter frustrations with false positives, information clutter, and interoperability issues (technology barriers), eroding trust (adopter-level barriers). Even when technical faults were later mitigated, these initial experiences limited or constrained uptake, illustrating how early technical and communication failures created enduring impressions that shaped subsequent patterns of trust and tool use.

This mutual reinforcement of challenges across domains aligns with the complexity perspective described by Greenhalgh et al [30] and Braithwaite et al [40], whereby interacting barriers within and across a complex adaptive system, such as health care, tend to compound rather than resolve over time, particularly when they are not addressed in a coordinated and simultaneous manner. Clinicians’ perceptions of value were shaped primarily by how reliably and efficiently the AI system performed within everyday reporting workflows. Early false positives and excessive image sets undermined those expectations, diminishing confidence in the tool’s promised efficiency benefits. This finding is consistent with a recent qualitative study showing that workflow fit determines perceived usefulness, even for AI [41]. Participants described the system as “a check-off” tool rather than an integrated aid, and acceptance was suboptimal across our study; consistent with a 2023 semistructured interview with radiologists (n=25), which identified reliability, interpretability, and feedback transparency as decisive for AI acceptance [14]. Even after performance improved, initial mistrust persisted. This enduring skepticism also mirrors broader evidence that initial experiences set adoption trajectories and that trust is far easier to lose than to regain [42]. The finding reinforces the importance of consolidating the first-use experience through predeployment testing or “shadow mode” configurations [43].

Organizational conditions were vital in shaping clinician engagement. During peri-implementation, fragmented communication and limited training left some staff unaware that the system had gone live, while others lacked the confidence to use it effectively. This mirrors the work of our team and others who have consistently highlighted that structured rollout, anticipatory planning, and capacity building are critical for sustainable digital adoption [32,44-47]. Participants described the process as reactive and isolated rather than coordinated, reflecting the absence of a shared sense of purpose and mutual accountability between leadership, implementers, and users, necessary for effective implementation [48]. Although clinicians remained receptive to AI, they expected visible organizational commitment through ongoing education, rapid troubleshooting, and coherent leadership. In its absence, they relied on informal workarounds and peer support to conduct work-as-done, the adaptive, improvised practices that frontline staff develop to keep systems functioning when formal processes or resources fall short [49]. While such adaptations can sustain local functionality, they also introduce variability in care delivery and make it difficult to scale and standardize best practices across settings.

These organizational shortfalls also shaped how clinicians experienced implementation. In the absence of clear planning and coordination, several described feeling individually responsible for interpreting and integrating the AI tool into their workflow. This was not an explicit transfer of responsibility but reflected a professional culture in which clinicians relied on their own judgement to make the system workable within local constraints. Medicolegal uncertainty about liability further reinforced this guarded engagement. A 2021 narrative review observed that when accountability is unclear or diffuse, clinicians maintain human oversight to protect both patient safety and professional authority [50]. These dynamics highlight a core implementation challenge: without clear institutional responsibility and evidentiary assurance, professional caution becomes self-reinforcing, constraining experimentation, shared learning, and the normalization of AI within routine practice.

The single-site public hospital context influenced organizational capacity. Frequent staff rotations and high turnover meant that many clinicians engaged with the AI system intermittently, limiting opportunities for cumulative learning. Similar patterns are common across public health services, where workforce mobility and resource constraints make it difficult to sustain iterative improvement [51]. These dynamics interacted with the implementation process itself, shaping the pace and pattern of adoption and modulating how performance issues were perceived over time. Rather than functioning as external confounders, they formed part of the organizational ecology within which the AI system was introduced, influencing continuity, familiarity, and the stability of feedback loops essential for embedding new technologies. These conditions underscore the importance of implementation approaches that are designed for continuity, including modular onboarding, periodic refresher training, and accessible repositories of AI-related resources to support learning across changing teams.

This study extends the AI-in-radiology literature in 3 key ways. It adopts a temporal perspective, tracing implementation from early anticipation to postintegration and showing how initial optimism and early missteps shape later engagement. It also demonstrates that adoption was driven not by the innovation alone but by the interaction of technical performance, organizational coordination, and context, extending NASSS from description to explanation. In this framing, staff turnover, workload fluctuations, and shifting operational priorities function as active contextual determinants that help explain why implementation trajectories evolve as they do, rather than as background noise. Finally, through reflexive use of the framework, the study generates theoretical insight into why implementation evolves as it does, contributing to emerging work on domain interdependence and temporal complexity in health care innovation [52].

Grounded in our findings and consistent with previous guidance [23,30,53,54], effective AI implementation in radiology depends on a combination of technical stability, communication, and organizational preparedness. Early “shadow mode” piloting helps identify faults and build trust before clinical use, while consistent communication about progress and fixes maintains transparency. Workflow-compatible design that minimizes cognitive load supports efficiency and acceptance [55]. Ongoing professional development, formal feedback channels with vendor responsiveness, and planning for workforce turnover through shared training repositories and local champions help sustain capability over time. Together, these strategies align with the six principles of FUTURE-AI recommendations encompassing fairness, universality, traceability, usability, robustness, and explainability, an international expert-driven consensus to facilitate the adoption of trustworthy medical imaging [56] and emphasize that co-design and iterative learning are essential to long-term adoption.

### Study Limitations and Strengths

This was a single-site study, and further real-world, ecological research is needed to identify determinants of adoption and create solutions that generalize across health systems. Despite this, our findings are similar to several recently published studies examining radiologists’ perceptions around the adoption of AI into standard practice [2,12,14,57-60]. There was low uptake of the tool during the peri-implementation period due to technical challenges. This limited wider evaluation of this crucial implementation period, particularly in terms of clinical usefulness. The 18-month period may have also introduced a range of system confounders, including staffing changes and organizational priorities, which may have impacted how participants felt about the AI clinical decision support tool. Finally, social desirability bias cannot be ruled out, particularly as this was a department-wide implementation. However, this was mitigated through participant briefings emphasizing the exploratory nature of the trial, coupled with reflexive practice by the interview team [61]. A key study strength was that this was a real-world evaluation demonstrating ecological validity with findings grounded in practical realities. Second, aligning our findings with a validated implementation science framework supports theoretical transferability and future application in related contexts despite the single-site limitations.

### Future Implications

Future studies should integrate qualitative and quantitative data, combining workflow observations with metrics such as reporting time, error rates, and AI usage logs, to triangulate findings. Multisite evaluations across differing levels of digital maturity are needed to test transferability and examine how governance, culture, and workforce patterns influence scalability.

### Conclusion

Implementation of AI-based decision support in radiology is as much an organizational and cultural process as a technological one. Clinicians remain willing to engage, but sustainable adoption depends on consolidating early experiences, embedding communication and training, and maintaining iterative feedback between users, vendors, and system leaders. Applying the NASSS framework revealed how domains interact dynamically across time, offering both theoretical insight into sociotechnical complexity and practical guidance for hospitals seeking to move from pilot to routine, trustworthy AI integration.

## Data Availability

The datasets generated or analyzed during this study are not publicly available due to confidentiality policies, but may be available in limited form from the corresponding author on reasonable request.

## Appendix

Multimedia Appendix 1 COREQ (Consolidated Criteria for Reporting Qualitative Studies) checklist.

Multimedia Appendix 2 Interview topic guide.

## Abbreviations

AI: artificial intelligence
CDSS: clinical decision support system
COREQ: Consolidated Criteria for Reporting Qualitative Studies
CT: computed tomography
ERIC: Expert Recommendations for Implementing Change
NASSS: nonadoption, abandonment, scale-up, spread, and sustainability
PACS: picture archiving and communication system
