# Supplementary material for: Implementing an Artificial Intelligence Decision Support System in Radiology: Prospective Qualitative Evaluation Study Using the Nonadoption Abandonment Scale-Up, Spread, and Sustainability (NASSS) Framework
Source: J Med Internet Res. 2026 Jan 28;28:e80342. doi: 10.2196/80342 (PMC12895160; doi:10.2196/80342)
Supplement: Multimedia Appendix 2 [file jmir_v28i1e80342_app2.docx]

**Understanding the implementation and use of AI-Rad Companion**

**How to use this guide:**

This interview guide is semi-structured by design and will be adapted for an individual and/or focus group context as appropriate. These questions will be adapted to either the pre- or post-implementation phase of this study and will refer to the most contextually relevant solutions being used by the participant (s) at the time of this interview.

The interviewer should follow the questions and prompts from the section(s) of the guide that most appropriately match the interviewees’ role(s). Within each section:

- - 1. All key questions should be asked
    2. Should other relevant issues be raised they should be explored using the prompts as required
    3. Interviewers will ensure discussion progresses in a timely, yet informative manner

**Introduction**

Hi, my name is [ ]. I’m speaking with you today because we are interested in understanding how clinical decision support systems like the implementation of the AI-RAD companion and related imaging support solutions technology are currently being used and implemented within your hospital. You have been selected as a key stakeholder, and we believe you have important information to share with us due to your role in [ insert role/team].

We will record the conversation with you if that is OK and will only share your conversation with other members of the research team or by making sure other people cannot identify your responses. We really appreciate your time and willingness to speak with us. Do you have any questions before we get started?

Do you agree to participate in this study?

Yes  No

Do you agree for non-identifiable data collected in this project to be used as comparative data for future studies related to this project?

Yes  No

Do you agree for non-identifiable data collected in this project to be used as comparative data for future studies that may not be related to this project?

Yes  No

**Context**

**1. Can you tell me about your role working within and/or collaborating with medical imaging services at PA hospital?**

Prompts:

- What multidisciplinary teams are you a part of?

- How much of your time is spent working directly with patients?

- How much of your time is spent in administration or management?

- How many staff do you manage?

**2. How would you describe your role as a decision maker/influencer in relation to any aspect of the medical imaging services at PA hospital?**

- Could unpack this process in greater detail for me
- Who else might be involved?
- Could you take me through this decision-making process from end to end as you perceive it?

**3. Are you familiar with the main imaging technologies currently used within the PAH medical imaging department? If so, how?**

Prompts

- What is the context of this engagement?

- What was your most recent example of this?

**4. What would you consider some of the current perceived barriers around the appropriate and effective utilisation of these tools more generally?**

Prompts

**-** Can you give me some concrete examples

- Why do you think this is the case?

- Has this changed over time?

- Probe the following factors

- Organisational?
- Clinical practice?
- Individual preferences?
- health service culture?
- Evidence base i.e. published literature/CPD

**Technology and Adopters**

5. **Delving further I'd like to talk about the AI-RAD companion tool, which has been piloted at PAH. Are you familiar with this decision support tool?**

Prompts

- If yes, probe how
- If no, probe why

**If yes to 5, unpack what they know about how and why this tool was implemented for piloting in the first place**

- Who was involved in making the key procurement decisions?
- Which factors influenced this?

**6. What are the key technical dependencies associated with implementing, using and/or supporting the use of AI-RAD companion?**

Prompts:

- Does the technology connect with existing infrastructure?
- Which other technology systems in the organisation this technology is linked to?
- Does it need to be installed across multiple technical systems to achieve ‘integration’?
- How do they all fit together?
- Will there need to be an upgrade to the organisation’s IT system (e.g. new hardware, better bandwidth) to support use of the technology across the organisation?

**7. How do you perceive the usability of the AI-RAD companion?**

Prompts:

- Do you personally use it as a part of your workflow routine?
- How does it compare to what you did before?
- Probe the following factors:
- Clinical processes
- Clinical outcomes
- Functionality
- Interface
- Learning curve
- Available support

**8. How do you think the implementation of AI-RAD has impacted upon service delivery for the department?**

Prompts:

- To what extent does implementing the technology require staff to do their jobs in a different way and/or interact with different people or teams?
- To what extent does implementing the technology require new or different steps in the care pathway (e.g. new administrative processes)?

**Probe- How has this impacted your workflow?**

**9. To what extent do you think the technology (and/or the service model it supports) will become obsolete or require updating in the next 3-5 years?**

**Prompts:**

- To what extent can the technology be adapted to take account of future changes?
- To what extent will the technology supply model change?

**Value Proposition**

**10.** **How would you describe/characterise the costs and benefits of this technology to patients?**

Prompts:

- Are there any high-quality studies (e.g. randomised controlled trials) to demonstrate the

technology’s efficacy for this patient/client group?

- What evidence is there that the technology’s benefits outweigh its potential harms?
- Have the technology’s efficacy and safety been measured in terms of an outcome that

matters to patients/clients

**11. How would you describe/characterise the costs and benefits of this technology to yourself as a [insert professional role]?**

Prompts:

- How would you describe its learning curve?
- How would evaluate its workflow impact?
- What are some of the intended and unintended consequences of its implementation in relation to your work?

**12. What is known about the value that this technology could bring to the health or care system?**

Prompts:

- Has the technology (or the technology-supported care model) been shown to have an overall

advantage over existing practice?

- Has technology been shown to be effective and cost-effective in terms of how much benefit it

will bring for a given financial outlay?

- Are there any safety concerns about the technology or the care model it supports? Are there concerns that the technology, whilst improving care for some patients, could result in an inequity of some outcomes?
- How would describe the regulatory environment that the AI-RAD companion and related technology are situated within?

**13. Is the value proposition likely to change over the next 3-5 years?**

Prompts:

- A new, better technology is on the horizon?
- The market for the technology could change significantly

**The organisation**

**14. To what extent do you think the work of implementing or adopting of this technology into practice has been realistically assessed and adequately resourced?**

Prompts:

- Work to bring people on board and develop a shared, organisation-wide vision for the

change?

- Work to develop, implement and mainstream new care pathways and processes?

- Work to coordinate the project across more than one organisation or sector?

- Work to evaluate and monitor the change?

**15. What have been some of the organisational barriers and enablers to implementing or adopting this technology into practice?**

Prompts:

- Leadership and decision-making processes
- Funding structures
- People and culture
- Regulatory environment
- Data access and stewardship

**16. How do you think the policy, regulatory and economic context for these innovations is likely to**

**change over the next 3-5 years? Is there likely to be turbulence?**

Prompts:

- Change of government?
- New policy priorities?
- Economic recession?
- New regulatory framework?
- Withdrawal of industry commitment

**17. Do you agree to being contacted by myself or another member of the research team to undergo a follow up interview within the next 18 months?**

[Will be captured in the recording and noted by interviewee on the PIS for the participant]

**[End of Interview]**
